# The assessment of smart city information security risk in China based on zGT2FSs and IAA method

**DOI:** 10.1038/s41598-022-07197-1

**Published:** 2022-02-28

**Authors:** Hui Zhao, Yiting Wang, Xin Liu

**Affiliations:** Qingdao University of Technology, Qingdao, China

**Keywords:** Applied mathematics, Information technology, Engineering, Mathematics and computing

## Abstract

The continuous expansion of the construction scale of smart city has reconstructed the urban information pattern. How to maintain the stability of information security while giving full play to the role of information sharing is a practical problem that must be solved for the sustainable development of smart city. Based on the information ecology theory, this paper construct the smart city information security risk evaluation system from six aspects. Then, zGT2FSs is established based on type-2 fuzzy set theory and IAA method, which fully considers the internal and external uncertainty of expert decision-making. According to the calculation results, the key influencing factors of information security risk of smart city are analyzed to provide suggestions and guidance for the formulation of information security control in the process of smart city construction in China.

## Introduction

Smart city deeply integrates emerging technologies such as big data, cloud computing and Internet of Things with urban construction^1,2^, which is conducive to the sustainable development of urban economy, society and environment, and at the same time, it has greatly changed the information pattern of smart city^3,4^, causing multi-angle impact on information security. For example, in the process of building smart city, there exists security risks such as hackers attacks and the lack of awareness of network security protection^5–7^. The Chinese government attaches great importance to the construction of smart city in urban development and governance. Vigorously promoting the construction of new smart city has become the strategic direction of China's urban development^8,9^. In the context of the massive urban data collection required for the prevention and control of the world's COVID-19, how to identify the key influencing factors of information security risks in smart city while giving full play to the great role of information sharing, collaboration and integration, and how to formulate and improve relevant policies to maintain information security and stability are practical problems that must be solved for the sustainable development of smart city.

Smart city aims to integrate all subsystems of the city by using advanced information technology and operate the city in a smarter way^10^. Compared to digital city, smart city empathizes people's subjective feelings, like care applications for the health emergency management and the vulnerable groups^11^, etc. At the technical level, smart city apply and integrate the latest information and communication technologies such as Internet of things and cloud computing, gathering many aspects of urban development such as sustainable, innovative and availability, and pursuing integration of ICT in transportation systems and many other systems in urban construction^12^; At the level of urban governance, smart city emphasize participatory governance, focusing on relationship between urban residents and local government by expanding the investment scale of human and social capital^13^; At the target level, smart city aim to realize the wisdom of urban governance, public services and people's life^14,15^.

At present, the academic research on smart city information security risks can be roughly divided into three categories: risk connotation, risk measurement and information security countermeasures. The risk research methods that have been applied to information security include Random Forest^16^, DEMATEL^17^, Bayesian Network^18^ and so on. Felipe^19^ proposed nine aspects of information security for smart city systems, including information access, information tracking, and cross-access, focusing on information security issues and countermeasures in the planning and implementation phases of smart city construction. Moch proposed a point of insightful view that in the process of smart city construction, local governments need to focus on the safety and security of urban planning, services and decision-making, which also requires the joint efforts of both urban residents and the government^20^. Wang Yin^21^ believed that the key reason for frequent smart city information security problems in China is that the construction is immature. In addition, due to the integration of technology, governance, manpower, external economy, society, ecological environment and other factors in the process of building a smart city, a large number of complex problems would arise^22^. The development and construction of smart city projects must go through the technology, safety and convenience evaluation and reach the target before acceptance, and the project construction should be based on planning, development and maintenance of land system, so as to ensure the balanced utilization of land in urban construction^23,24^. Among the existing research results, most risk studies are only considered from the perspective of information technology risks, and the results of holistic analysis from multiple angles are few. Moreover, the uncertainty in decision-making is seldom considered when determining the indicator weight, which includes the inter-uncertainty when experts make decisions, and the intra-uncertainty when experts make decisions on the same object at different times.

Higher order fuzzy logic systems such as interval type-2 fuzzy logic systems have been shown to be very well suited to dealing with the large amounts of uncertainties present in the majority of real world applications^25,26^. Type-2 fuzzy set (T2 FSs), as a three-dimensional fuzzy set, has a better ability to measure fuzziness than type-1 fuzzy set (T1 FSs) in two-dimensional space. Inter and intra uncertainties can be identified and modeled using the different degrees of freedom of type-2 FSs, thus providing a clear representation and separation of these individual types of uncertainty present in the data^27–29^. ZSlice method of T2 FSs reduce both the complexity and the computational requirements for general type-2 fuzzy logic systems. As well as IAA method based on zSlice-based general type-2 fuzzy sets (zGT2FSs) can well measure the inter and intra uncertainties in decision making and make the evaluation process more accurate and comprehensive^30^.

In this paper, information security risk assessment system of smart city is established based on the information ecology theory, and IAA method based on zGT2FSs is used to calculate the index weights, providing reference for the related research and decision-making of information security risk of smart city.

## Materials and methods

### zSlice-based general type-2 fuzzy sets (zGT2FSs)

#### Type-1 fuzzy sets (T1 FSs) and Type-2 fuzzy sets (T2 FSs)

Classical Logic (represented by Boolean logic) holds that all objects or statements can be represented by binary terms such as 0 or 1, yes or no, black or white^31^. Given the set $X$, every element in its universe either belongs to the set $X$ completely or does not belong to $X$ at all, and there is no case where part of it belongs to $X$. However, the semantic concepts used in people's daily communication are often uncertain, and whether an element belongs to a semantic concept is often a gradual process rather than a sudden change, which cannot be simply described by the yes and no^32,33^.

**Figure 1:**
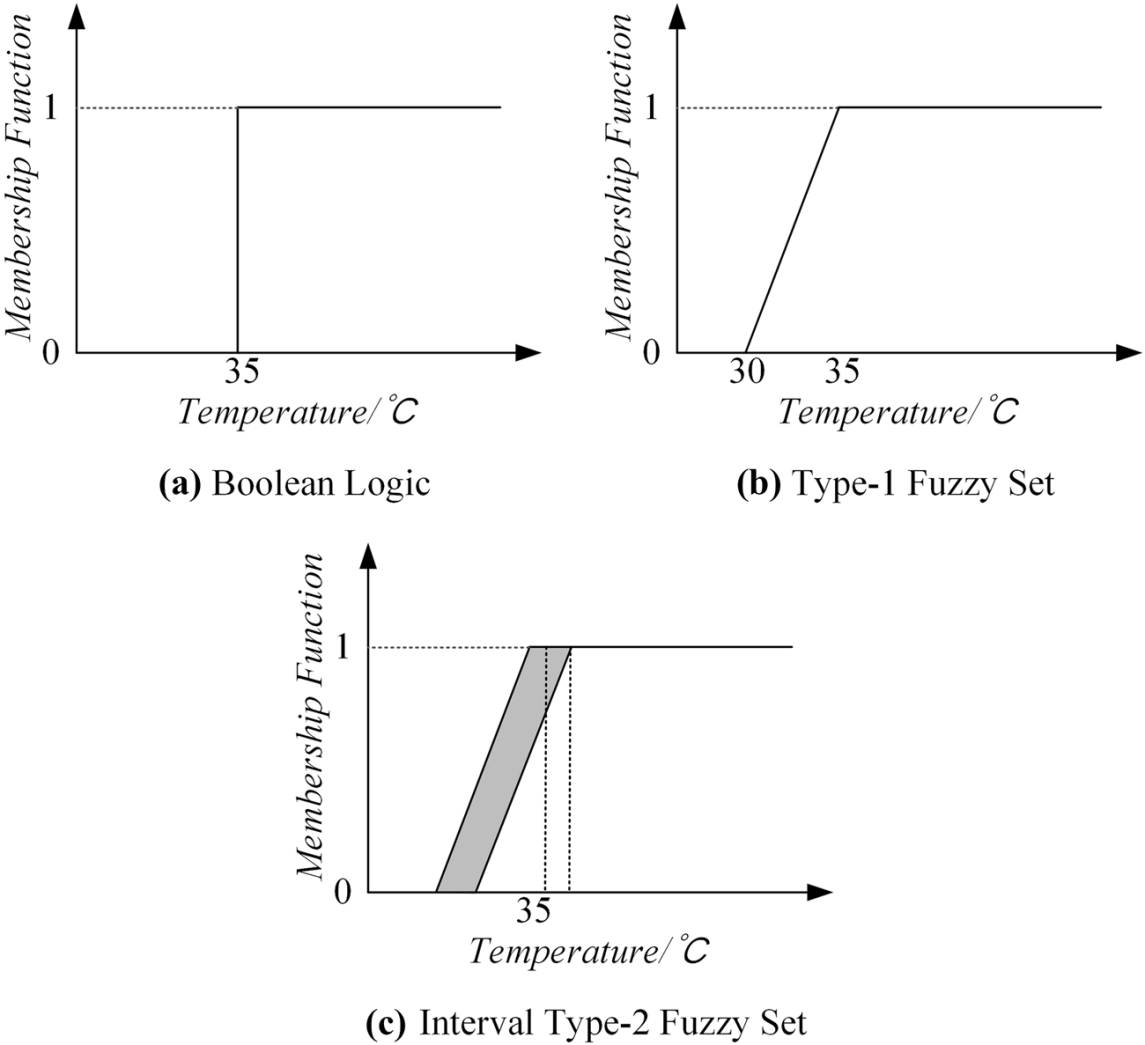
Different representations of temperature by Boolean logic (classic logic), T1 FSs and IT2 FSs.

In order to better model semantic concepts, Zadeh^34^ put forward fuzzy set theory (as T1 FSs in this paper). Compared with classical logic, T1 FSs can better measure the uncertainty of a single user's understanding of semantic concepts, which is Intra uncertainty. In 1975, Zadah^35^ put forward the concept of T2 FSs based on T1 FSs. Compared with T1 FSs, T2 FSs are characterized by 3 dimensional MFs, which in turn making T2 FSs a better ways to solve the high levels of uncertainty^36^. Different Representations of Temperature By Boolean Logic (Classic Logic), T1 FSs and IT2 FSs are shown in Fig. 1.

A T1 FSs can be generalized as as set function on a universe $X$ into $\left[ {0,1} \right]$. MF can be represented by $\mu \left( x \right)$ and classic T1 FS can be defined as:

1 $$A = \left\{ {\left( {x,\mu_{A} \left( x \right)} \right)\left. {} \right|\forall x \in X} \right\}$$

where $X$ is continues, $A$ can be commonly formalized as:

2 $$A = \int {_{x} \mu_{A} \left( x \right)/x}$$

where $\int {}$ is union over all $x \in X$.

The T2 FSs can be defined as:

3 $$\tilde{A} = \left\{ {\left. {\left( {\left( {x,u} \right),\mu_{{\tilde{A}}} \left( {x,u} \right)} \right)} \right|\forall x \in X,\forall u \in J_{x} \subseteq \left[ {0,1} \right]} \right\}$$

Or

4 $$\tilde{A} = \int {_{x \in X} \int {_{{u \in J_{x} }} {{\mu_{{\tilde{A}}} \left( {x,u} \right)} \mathord{\left/ {\vphantom {{\mu_{{\tilde{A}}} \left( {x,u} \right)} {\left( {x,u} \right)}}} \right. \kern-\nulldelimiterspace} {\left( {x,u} \right)}}} }$$

where $J_{x}$ is the primary membership and $J_{x} \in \left[ {0,1} \right]$, $\mu_{{\tilde{A}}} \left( {x,u} \right)$ is the secondary membership corresponding to each primary membership and $0 \le \mu_{{\tilde{A}}} \left( {x,u} \right) \le 1$.

#### Interval Type-2 fuzzy sets (IT2 FSs)

**Figure 2:**
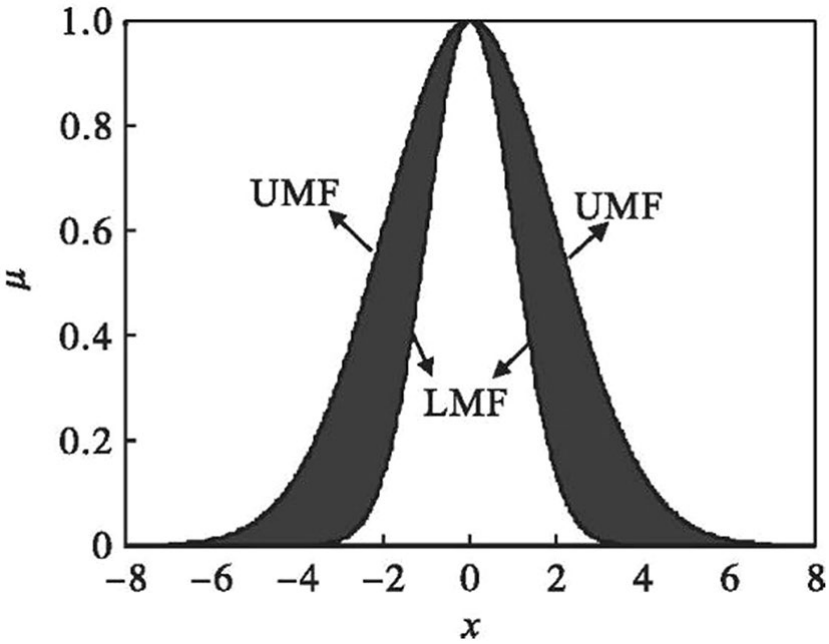
Membership function of IT2 FSs.

Interval Type-2 Fuzzy Sets (IT2 FSs) are simplification forms of T2 FSs which the primary membership is defined as the interval $\left[ {\underline{y} ,\overline{y} } \right]$, where $\underline{y}$ and $\overline{y}$ represent the different degrees of membership of $x$ in the lower membership function (LMF) and upper membership function (UMF) respectively^30,37^, we give a sample of membership function of IT2 FSs in Fig. 2.

Thus, a IT2 FS can be presented as:

5 $$\tilde{A} = \int_{x \in X} {\int_{{y \in \left[ {\underline{y}_{x} ,\overline{y}_{x} } \right]}} {1/\left( {x,y} \right)} }$$

IT2 FSs also can be expressed as:

6 $$\tilde{A} = \left\{ {\left( {x,A\left( x \right) = \left[ {\underline{A} \left( x \right),\overline{A} \left( x \right)} \right]} \right)|x \in X} \right\}$$

where the mapping $A:X \to L\left( {\left[ {0,1} \right]} \right)$ is the membership function, $\underline{A} \left( x \right)$ and $\underline{A} \left( x \right)$ represent the LMF and UMF. And it is worth noting that $L\left( {\left[ {0,1} \right]} \right)$ represents all close subinterval of $\left[ {0,1} \right]$, expressed as:

7 $$L\left( {\left[ {0,1} \right]} \right) = \left\{ {X^{\prime} = \left[ {\underline{x} ,\overline{x} } \right]|\left( {\underline{x} ,\overline{x} } \right) \in \left[ {0,1} \right]^{2} ,\underline{x} \le \overline{x} } \right\}$$

LMF, UMF and FOU (Footprint of Uncertainty) can be written as:

8 $$UMF = \left\{ {\left( {x,b_{x} } \right),x \in X} \right\}$$

9 $$LMF = \left\{ {\left( {x,a_{x} } \right),x \in X} \right\}$$

10 $$FOU = \bigcup\limits_{x \in X} {x \times L_{x} } = \bigcup\limits_{x \in X} {x \times \left[ {a_{x} ,b_{x} } \right]}$$

#### GT2 FSs based on zSlices (zGT2FSs)

In respect to the set operation of T2 FSs, Karnik present the Vertical Slices^38^ to facilitate the discretized point of T2 FSs where at each $x$ exist a secondary membership, and defined the secondary membership as Vertical Slice. Since this method is intuitive and easy to understand, it has been widely popularized and applied^39,40^. The vertical slice based on T2 FSs in the universe $X$ can be expressed as:

11 $$\tilde{A} = \int_{{x^{\prime} \in X}} {\mu_{{\tilde{A}}} \left( {x^{\prime}} \right) = \int_{{x^{\prime} \in X}} {\left[ {\int_{{u^{\prime} \in J_{{x^{\prime}}} }} {f_{{x^{\prime}}} \left( {u^{\prime}} \right)/u^{\prime}} } \right]} } ,\quad 0 \le f_{{x^{\prime}}} \left( {u^{\prime}} \right) \le 1,\quad J_{{x^{\prime}}} \subseteq \left[ {0,1} \right]$$

On the basis of vertical slice, scholars put forward wavy slices^41^, computational geometry approach^42^ and other methods^43^.

**Figure 3:**
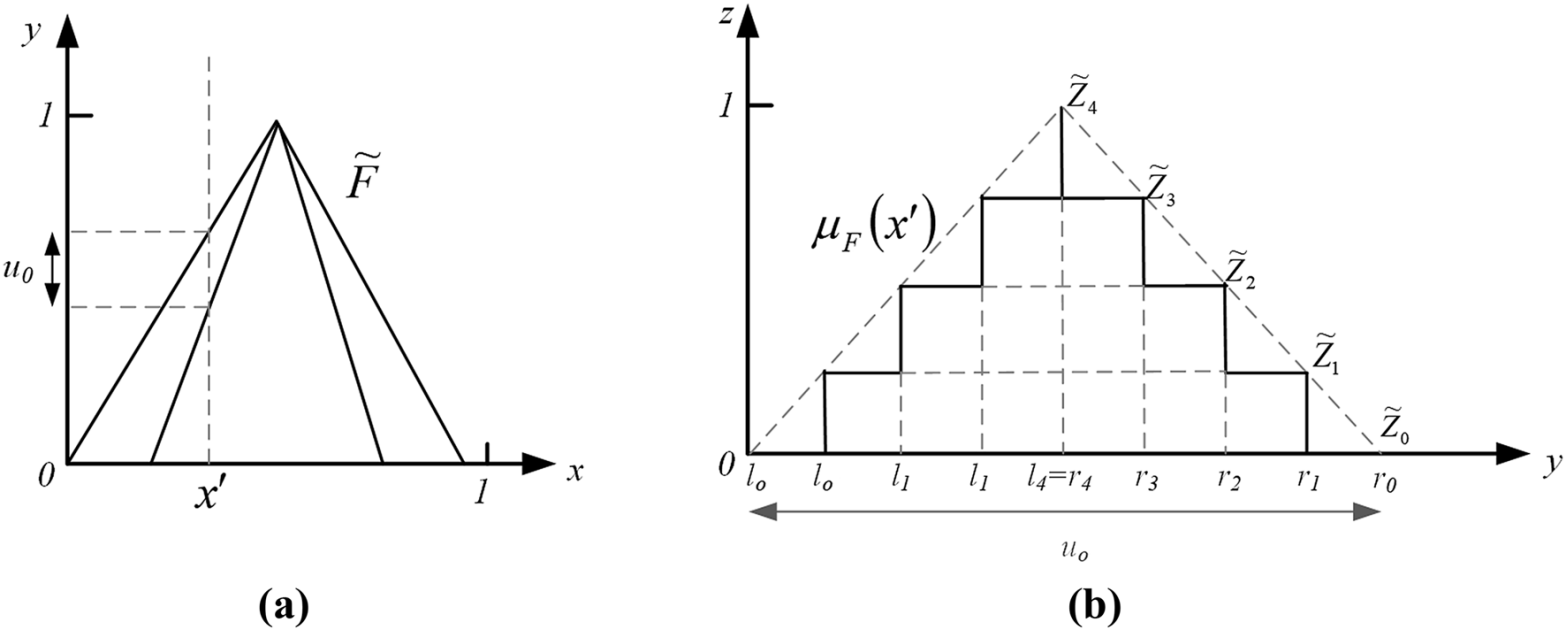
**(a)** Front view of a general type-2 set $\tilde{F}$. **(b)** Third dimension at $x^{\prime}$ of a zSlice based type-2 fuzzy set.

A zSlice is formed by slicing a general type-2 fuzzy set in the third dimension (*z*) at level $z_{i}$, creating an interval set with height $z_{i}$ in the third dimension (as Fig. 3). A zSlice $\tilde{Z}_{i}$ can be expressed as:

12 $$\tilde{Z}_{i} = \int {_{x \in X} \int {_{{u_{i} \in J_{{i_{x} }} }} {{z_{i} } \mathord{\left/ {\vphantom {{z_{i} } {\left( {x,u_{i} } \right)}}} \right. \kern-\nulldelimiterspace} {\left( {x,u_{i} } \right)}}} }$$

Or

13 $$\tilde{Z}_{i} = \int {_{x \in X} \int {_{{u_{i} \in \left[ {l_{i} ,r_{i} } \right]}} {{z_{i} } \mathord{\left/ {\vphantom {{z_{i} } {\left( {x,u_{i} } \right)}}} \right. \kern-\nulldelimiterspace} {\left( {x,u_{i} } \right)}}} }$$

Or

14 $$\tilde{Z}_{i} = \left\{ {\left. {\left( {\left( {x_{i} ,u_{i} } \right),z_{i} } \right)} \right|\forall x \in X,\forall u_{i} \in \left[ {l_{i} ,r_{i} } \right]} \right\}$$

where $z_{i} = i/I$,$1 \le i \le I$, the notation $I$ means the the number of zSlices. In Eq. (11), a zSlice $\tilde{Z}_{i}$ is equal to a T2 FSs which membership grade $\mu_{{\tilde{Z}_{i} \left( {x,u} \right)}}$ in the third dimension equal $z_{i}$,$0 \le z_{i} \le 1$.

Specially, when $$z = 0$$,

15 $$\tilde{Z}_{0} = \int {_{x \in X} \int {_{{u_{i} \in J_{x} }} {0 \mathord{\left/ {\vphantom {0 {\left( {x,u} \right)}}} \right. \kern-\nulldelimiterspace} {\left( {x,u} \right)}}} }$$

A general T2 FS $\tilde{F}$ is equal to the collection of zSlices:

16 $$\tilde{F} = \int {_{0 \le i \le I} \tilde{Z}_{i} } ,I \to \infty$$

In a discrete situation, Eq. (15) can also be written as:

17 $$\tilde{F} = \sum\nolimits_{0 \le i \le I} {\tilde{Z}}_{i}$$

The MF $\mu_{{\tilde{G}}} \left( {x^{\prime}} \right)$ of the zSlice based general type-2 fuzzy set (zGT2FSs)$\tilde{F}$ can be written as:

18 $$\mu_{{\tilde{F}}} \left( {x^{\prime}} \right) = \int {_{{\mu \in J_{{x^{\prime}}} }} \max \left( {z_{i} } \right)/u,J_{{x^{\prime}}} } \subseteq \left[ {0,1} \right]$$

where $0 \le i \le I$,$\mu_{{\tilde{G}}} \left( {x^{\prime}} \right)$ is a T1 FS.

### Interval agreement approach (IAA)

The academic method research on modeling survey based data using T2 FSs such as the interval approach (IA)^44^ and enhanced interval approach (EIA)^45,46^ has made a lot of progress, but these methods require data preprocessing and specific FSs forms, which makes it difficult for calculation and operation^47^. Wagner (2014)^48^ proposed a new approach about how uncertain intervals (where there is uncertainty about the endpoints of intervals) collected from decision-makers or multiple survey participants over repeated surveys can be modeled using type-1, interval type-2, or general type-2 FSs based on zSlices, named interval agreement approach (IAA). This method captures and models survey-based uncertainty requiring no data preprocessing and the prior definition of a specified MT type^49^.

IAA method can effectively reduce the quantity and degree of assumptions. In addition, this method constructs nonparametric model based on interval data without determining the specific type of FSs (such as Gaussian and Triangular)^50,51^. At same times it can greatly diminish the loss of information when reduce the higher ordered model^52^.

In this paper, we focus on modeling more uncertainty intervals from multiple sources, therefore, we only explain the principle in that case. And it is worth note that we claim all methods were carried out in accordance with relevant guidelines and regulations, all experimental protocols were approved by the Academic Ethics Committee of Qingdao University of Technology, Academic Committee of Qingdao University of Technology, informed consent was obtained from all subjects and/or their legal guardians. IAA method is conducted by following steps.

*Step 1*. Generate the IT2 FSs for each source.

19 $$\mu \left( A \right) = y_{1} / \cup_{{i_{1} = 1}}^{N} \overline{A}_{i} + y_{2} / \cup_{{i_{1} = 1}}^{N - 1} \cup_{{i_{2} = i_{1} + 1}}^{N - 1} \left( {\overline{A}_{{i_{1} }} \cap \overline{A}_{{i_{2} }} } \right) + \cdots + y_{N} /\left( { \cup_{{i_{1} = 1}}^{1} \cdots \cup_{{i_{N} = N}}^{N} \left( {\overline{A}_{{i_{1} }} \cap \cdots \cap \overline{A}_{{i_{N} }} } \right)} \right)$$

where $y_{i} = \frac{i}{N}$. And it is worth noting that we employ Eq. (18) independently for all outer and inner endpoints, create the UMF and LMF of IT2 FSs model.

*Step 2.* Aggregate IT2 FSs to create a zGT2 FS.

20 $$\mu \left( {\tilde{A}} \right) = z_{1} / \cup_{{i_{1} = 1}}^{N} A_{{i_{1} }} + z_{2} / \cup_{{i_{1} = 1}}^{N - 1} \cup_{{i_{2} = i_{1} + 1}}^{N - 1} \left( {A_{{i_{1} }} \cap A_{{i_{2} }} } \right) + \cdots + z_{N} /\left( { \cup_{{i_{1} = 1}}^{1} \cdots \cup_{{i_{N} = N}}^{N} \left( {A_{{i_{1} }} \cap \cdots \cap A_{{i_{N} }} } \right)} \right)$$

where $z_{i} = \frac{i}{N}$. In this step, Eq. (19) is applied twice to get all source-specific UMFs and LMFs resulting in the UMFs and LMFs of respective zSlices.

The steps above create zGT2FS that provides a model of both intra and inter uncertainty for the given set of uncertain intervals.

## Case study

### Problem description

**Figure 4:**
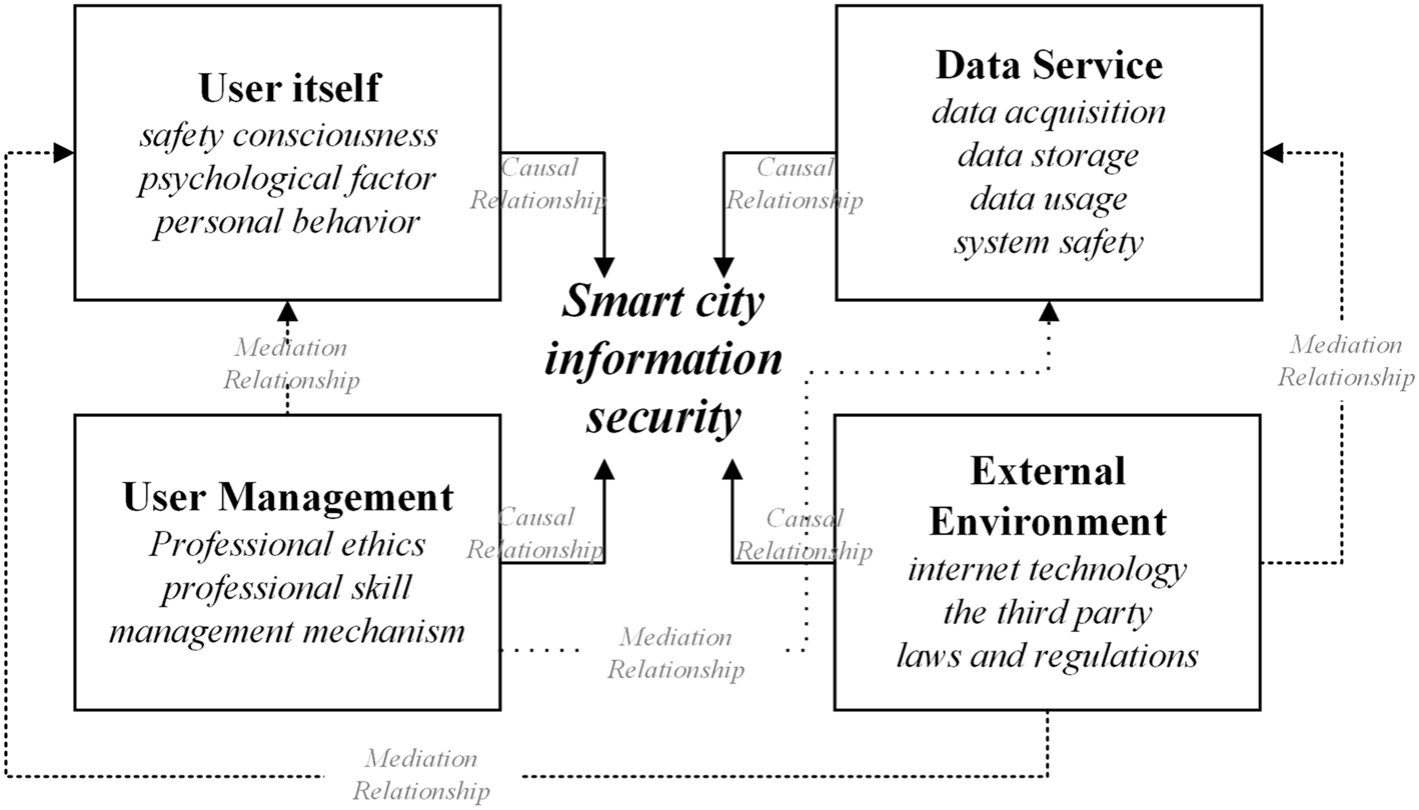
Smart city information security impact framework.

The application of emerging technologies in the construction of smart city fully integrates data resources and changes the information pattern of cities, leading to significant changes in the connotation and conformation of information security of smart city, bringing about a multi-faceted impact on information security (Fig. 4). Information security is the foundation of smart city construction and the guarantee of healthy development of it, hence the significance for the information security risk evaluation to ensure sustainable development. In China, promote the construction of new smart city has become the strategic direction of China's urban development^53^. With the gradual deepening of smart city construction, the issue of information security has become an increasingly prominent focus^54^. Information security is the foundation of smart city construction and the guarantee of healthy development of smart city^55^, which plays a vital role in smart city system and even national and social stability.

The problem faced by the decision makers in China's smart city project is to prioritize the dimensions and application areas separately so that project resources are allocated according to the importance and urgency of the each application area and the dimension related with it. The application of the smart city concept is conceptualized as a MCDM problem and IAA approach with zSlice type-2 fuzzy sets is utilized to solve this problem.

### Smart city information security risk evaluation indicator system

**Figure 5:**
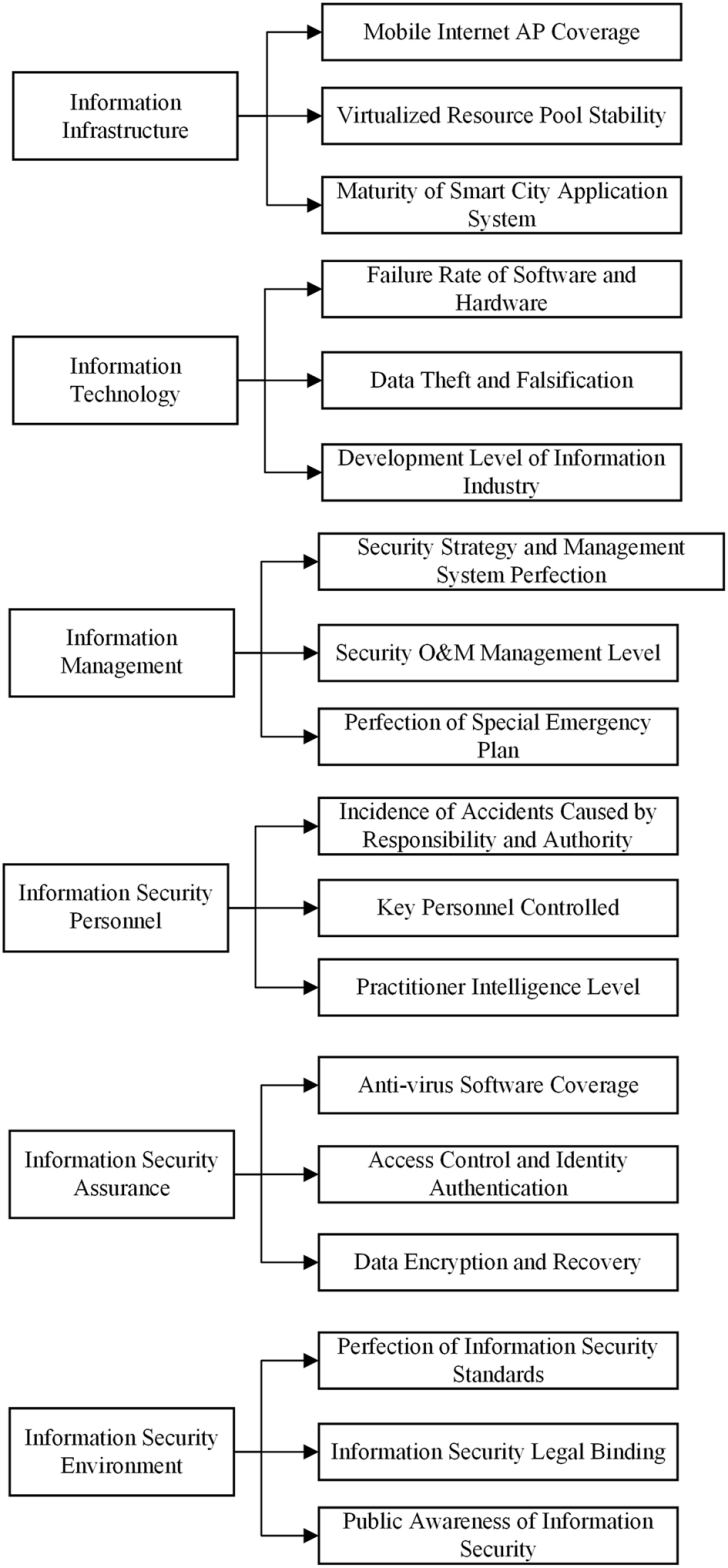
Smart city information security risk evaluation indicator system.

In 1869, Haeckel^56^ put forward the concept of ecology for the first time, he stated that ecology is the whole relationship between animals, organic and inorganic environments. Ecology developed rapidly and penetrated into many disciplines after that^57–61^. Information ecology is a new subject research field which emerged from the intersection of information science and ecology, existing studies have confirmed the applicability of information ecology theory in information security system^62–64^. Smart city is a typical information ecosystem which covers many elements such as people, information, technology and institutions. Based on the information ecology theory, we investigate the elements of smart city information ecosystem and existing security risks, comprehensively analyze the characteristics of information security risks in smart city according to the roles and influences among the elements and construct the evaluation indicator system. Through analyzing the literature in recent 10 years and screening the indicators (details at ESM Appendix I), the evaluation indicator system of information security risk assessment is shown in Fig. 5.

### Experimental results

Aiming at the information security risk evaluation of smart city, four experts were selected from different organizations for interview research. The purpose of this study is to provide decision-making reference for professionals who are responsible for information security risks in smart city. Therefore, we do not cover all stakeholders of information security risks in smart city in an all-round way, but give priority to the opinions of the implementation and decision-makers who have a say in the project. The four experts selected in this paper have more than 10 years of relevant experience in their specific fields in smart city and have decision-making ability in their organizations.

**Figure 6:**
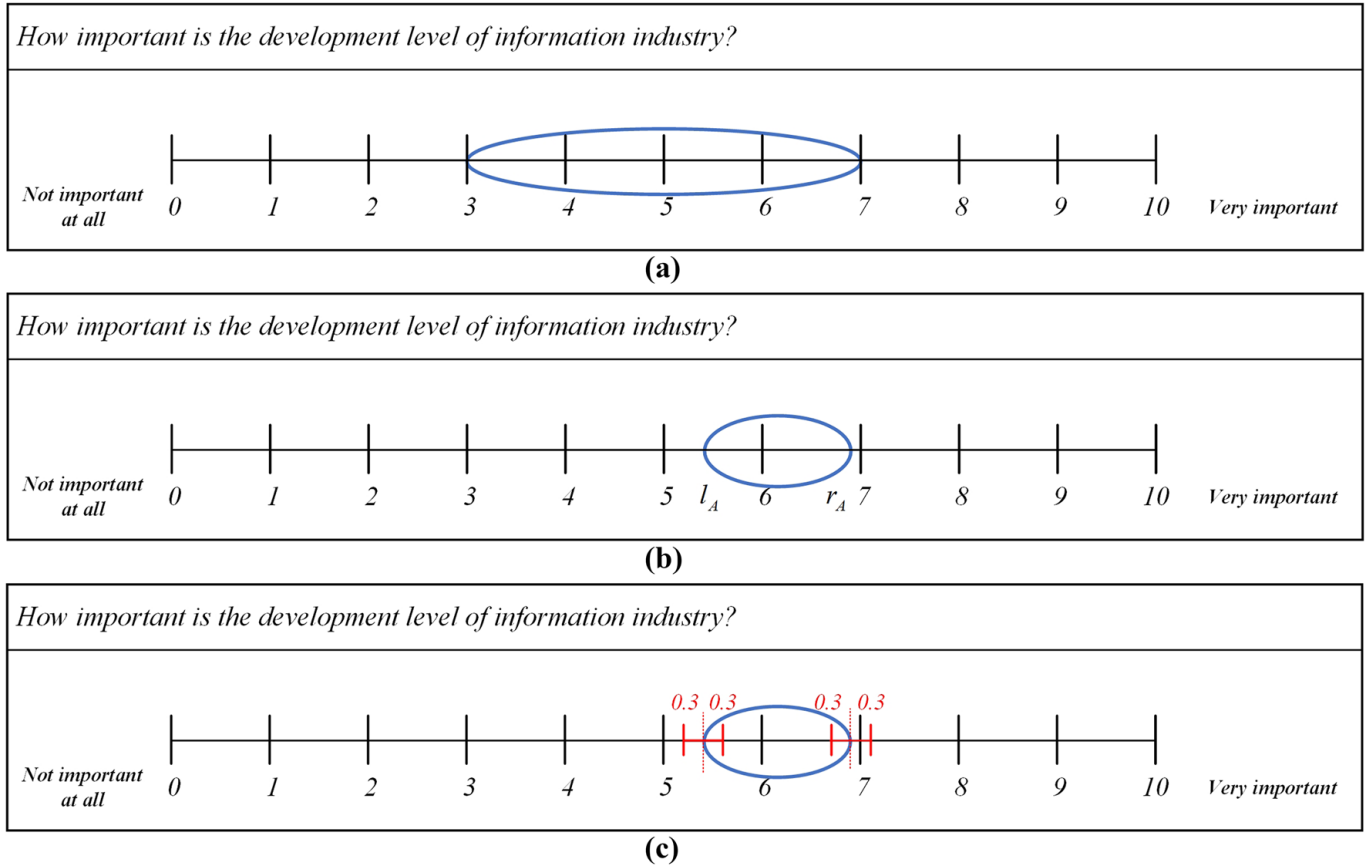
Example interval response (where **(a)** represents a more uncertainty response, **(b)** represents a less uncertainty response, **(c)** shows the uncertain intervals where for the latter each endpoint is itself an interval (variance)).

According to IAA, in order to capture uncertainty during data collection, the survey design in which experts can express their uncertainty about a given response by specifying an interval, rather than specifying or choosing a crisp point such as on Likert scale. Each decision-makers is asked to provide a variance in each decision which can be interpreted as the uncertainty of in their answers. In this method, experts’ certainty in their view denoted by the width of the interval, a narrow interval indicates that experts are sure about their answer, a wider interval means that they are less certain (as Fig. 6).

In this paper, three repeated questionnaires were conducted for four experts at different 3 times. The contents of the questionnaires were the same, and a total of 12 questionnaire results were obtained, based on which the internal and external uncertainties in the expert decision-making process were measured. Each pair of intervals is calculated according:

21 $$\ddot{p} = \left[ {\left[ {a - u,a + u} \right],\left[ {b - u,b + u} \right]} \right]$$

where $\ddot{p}$ is the resulting pair of intervals, $u$ is the uncertainty value, and $\left[ {a,b} \right]$ is an expert’s opinion.

In order to show the above decision-making process more intuitively, the ellipse drawn by experts from one of the surveys is summarized in digital form as shown in Table 1.

**Table 1:** One of the expert decision result.

Indicators	Expert A		Expert B		Expert C		Expert D	
	Answer	Uncertainty	Answer	Uncertainty	Answer	Uncertainty	Answer	Uncertainty
Mobile internet AP coverage	4–7	0.5	5–6.5	0.2	4–6	1.1	4.5–6.5	0.4
Virtualized resource pool stability	5–7	0.5	3–6	0.9	4–7	0.3	5.5–6.5	0.9
Maturity of smart city application system	6–8	1	5–8	0.5	6–9	0.2	4–6	1.5
Failure rate of software and hardware	6–9	0.8	5–7.5	1.5	6–8	0.5	6.5–7.5	0.7
Data theft and falsification	2–7.5	0.5	4–6	0.5	3–6	0.8	2–5	0.2
Development level of information industry	4–6	1.6	4–7	0.3	5–9	0.5	7–8	0.4
Security strategy and management	2–5	0.3	3–5	0.5	2–4	0.2	3–4	0.7
Security O&M management level	2–8	0.5	3–8.5	0.5	2.5–7.5	0.5	4–6.5	0.6
Perfection of special emergency plan	2–6	0.2	4–5	1.1	3–5	0.6	3–6	0.3
Incidence of accidents caused by responsibility and authority	5–7	0.4	4–6	0.5	3–5	0.9	5–8	0.1
Key personnel controlled	4–6.5	0.8	3–7	0.1	5–6	0.4	4–6.5	0.5
Practitioner intelligence level	6–9	0.2	5–8	0.5	7–8	0.5	6–7	0.3
Anti-virus software coverage	3–6	0.6	4.5–7	0.2	6–7	0.1	4–5	1.5
Access control and identity authentication	5.5–8	0.5	6–8	0.5	5–7	0.3	6–7.5	0.4
Data encryption and recovery	7–9	1.2	6–9	0.6	5–8	0.2	7–8	0.6
Perfection of information security standards	4.5–5	0.8	3–6	0.1	4–7.5	0.4	5–6	0.8
Information security legal binding	3–4	0.5	2–5	0.5	5–6	0.5	2–4	0.7
Public awareness of information security	7–9	1	5–9.5	0.2	6–8	0.3	7–8	1.5

Next, calculate the intervals according to Eq. (20), the results are showed as Table 2.

**Table 2:** Uncertain intervals of one expert conducted from the expert decision result of the indicator security O&M management level.

Experts	1st survey	2nd survey	3rd survey
A	$\left[ {\left[ {0.15,0.25} \right],\left[ {0.75,0.85} \right]} \right]$	$\left[ {\left[ {0.25,0.35} \right],\left[ {0.80,0.90} \right]} \right]$	$\left[ {\left[ {0.20,0.30} \right],\left[ {0.70,0.80} \right]} \right]$
B	$\left[ {\left[ {0.25,0.35} \right],\left[ {0.80,0.90} \right]} \right]$	$\left[ {\left[ {0.22,0.28} \right],\left[ {0.77,0.83} \right]} \right]$	$\left[ {\left[ {0.31,0.39} \right],\left[ {0.76,0.84} \right]} \right]$
C	$\left[ {\left[ {0.20,0.30} \right],\left[ {0.70,0.80} \right]} \right]$	$\left[ {\left[ {0.14,0.26} \right],\left[ {0.74,0.86} \right]} \right]$	$\left[ {\left[ {0.35,0.45} \right],\left[ {0.65,0.75} \right]} \right]$
D	$\left[ {\left[ {0.34,0.46} \right],\left[ {0.59,0.71} \right]} \right]$	$\left[ {\left[ {0.35,0.45} \right],\left[ {0.62,0.72} \right]} \right]$	$\left[ {\left[ {0.31,0.39} \right],\left[ {0.66,0.74} \right]} \right]$

Using Eq. (18) with the above intervals (detailed calculations can be found in ESM Appendix II, (2)), results in

22 $$\overline{\mu }\left( {\tilde{A}} \right) = \left( {y_{1} /\left[ {0.15,0.90} \right] + y_{2} /\left[ {0.20,0.85} \right] + y_{3} /\left[ {0.25,0.80} \right]} \right)$$

23 $$\underline{\mu } \left( {\tilde{A}} \right) = \left( {y_{1} /\left[ {0.25,0.80} \right] + y_{2} /\left[ {0.30,0.75} \right] + y_{3} /\left[ {0.35,0.70} \right]} \right)$$

It is worth noting that the notation $\overline{\mu }\left( {\tilde{A}} \right)$ means the UMF and $\underline{\mu } \left( {\tilde{A}} \right)$ means the LMF which together completely describe the IT2 FS $\tilde{A}$ for expert A.

After complete generating the IT2 FSs, we proceed to step 2 to create a zGT2 FS that can representing the intra and inter uncertainty. According to “Materials and methods”, the secondary membership domain is divided into 4 levels, at membership degrees of 0.25, 0.5, 0.75 and 1, also can be express as $\tilde{Z}_{1} = 1/4 = 0.25$, $\tilde{Z}_{2} = 2/4 = 0.5$, $\tilde{Z}_{3} = 3/4 = 0.75$ and $\tilde{Z}_{4} = 1$. Equations (24) and (25) give the details of $\tilde{Z}_{1}$ which are calculated using Eq. (19), and a more detailed view of the calculations can be found in ESM Appendix II.

$$\tilde{Z}_{{\overline{1}}} = 0.25/\left( {\left( {0.33/\left[ {0.14,0.90} \right]} \right) + \left( {0.66/\left[ {0.20,0.85} \right]} \right) + \left( {1/\left[ {0.25,0.83} \right]} \right)} \right)$$

$$\tilde{Z}_{{\underline{1} }} = 0.25/\left( {\left( {0.33/\left[ {0.25,0.80} \right]} \right) + \left( {0.66/\left[ {0.30,0.77} \right]} \right) + \left( {1/\left[ {0.35,0.76} \right]} \right)} \right)$$

$$\tilde{Z}_{{\overline{2}}} = 0.5/\left( {\left( {0.33/\left[ {0.15,0.90} \right]} \right) + \left( {0.66/\left[ {0.20,0.84} \right]} \right) + \left( {1/\left[ {0.31,0.80} \right]} \right)} \right)$$

$$\tilde{Z}_{{\underline{2} }} = 0.5/\left( {\left( {0.33/\left[ {0.26,0.80} \right]} \right) + \left( {0.66/\left[ {0.30,0.75} \right]} \right) + \left( {1/\left[ {0.39,0.70} \right]} \right)} \right)$$

$$\tilde{Z}_{{\overline{3}}} = 0.75/\left( {\left( {0.33/\left[ {0.22,0.86} \right]} \right) + \left( {0.66/\left[ {0.25,0.80} \right]} \right) + \left( {1/\left[ {0.35,0.75} \right]} \right)} \right)$$

$$\tilde{Z}_{{\underline{3} }} = 0.75/\left( {\left( {0.33/\left[ {0.28,0.74} \right]} \right) + \left( {0.66/\left[ {0.35,0.70} \right]} \right) + \left( {1/\left[ {0.45,0.65} \right]} \right)} \right)$$

$$\tilde{Z}_{{\overline{4}}} = 1/\left( {\left( {0.33/\left[ {0.31,0.74} \right]} \right) + \left( {0.66/\left[ {0.34,0.72} \right]} \right) + \left( {1/\left[ {0.35,0.71} \right]} \right)} \right)$$

24 $$\tilde{Z}_{{\underline{4} }} = 1/\left( {\left( {0.33/\left[ {0.39,0.66} \right]} \right) + \left( {0.66/\left[ {0.45,0.62} \right]} \right) + \left( {1/\left[ {0.46,0.59} \right]} \right)} \right)$$

25 $$\tilde{Z} = \tilde{Z}_{1} \cup \tilde{Z}_{2} \cup \tilde{Z}_{3} \cup \tilde{Z}_{4}$$

**Figure 7:**
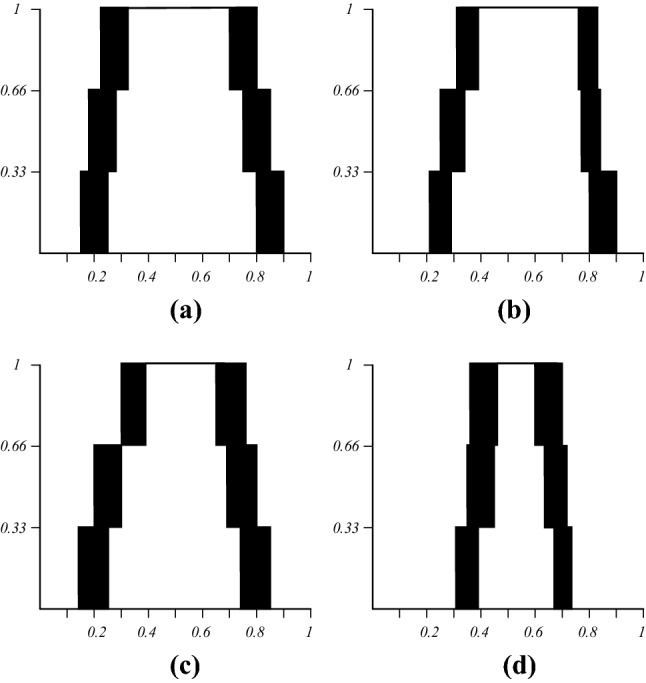
IT2 FSs produced with IAA **(a)** Expert A, **(b)** Expert B, **(c)** Expert C, **(d)** Expert D.

**Figure 8:**
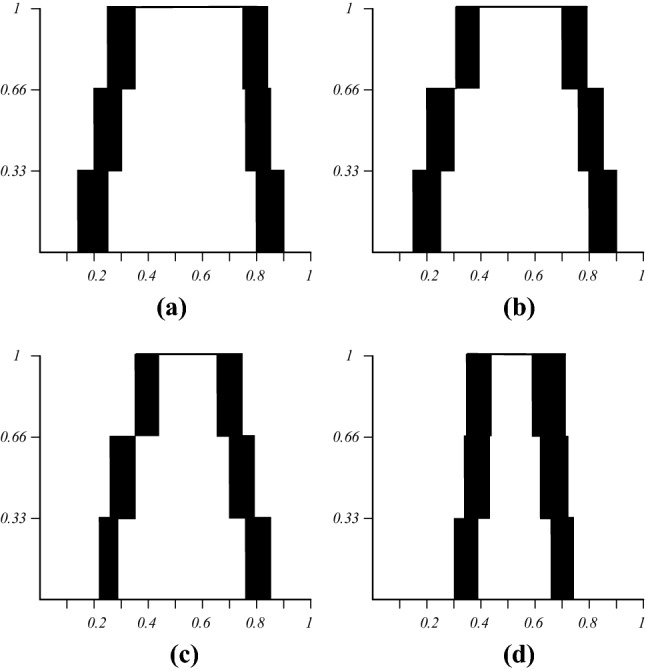
GT2 FSs $\tilde{Z}_{i}$ produced with IAA. **(a)** $\tilde{Z}_{1} = 0.25$, **(b)** $\tilde{Z}_{2} = 0.5$, **(c)** $\tilde{Z}_{3} = 0.75$, **(d)** $\tilde{Z}_{4} = 1$.

The IT2 FSs and zGT2 FSs based on expert decision results constructed by IAA method are obtained through the above steps, as shown in Figs. 7 and 8. Figure 7 shows the IT2 FSs created for each of the experts over the three surveys, Fig. 8 shows the zSlices at the respective secondary membership degrees (zLevels) of 0.25, 0.5, 0.75, and 1.

Repeat the above steps to obtain zGT2FSs corresponding to 18 indicators. The calculation results are summarized in Table 3. Thus, the indicator weights are showed in Table 4.

**Table 3:** zSlice details with intervals and associated primary and secondary membership.

	$\tilde{Z}_{1} = 0.25$	$\tilde{Z}_{2} = 0.5$	$\tilde{Z}_{3} = 0.75$	$\tilde{Z}_{4} = 1$
UMFs	$\tilde{Z}_{{\overline{1}}}$	$\tilde{Z}_{{\overline{2}}}$	$\tilde{Z}_{{\overline{3}}}$	$\tilde{Z}_{{\overline{4}}}$
$y = 0.33$	$\left[ {0.14,0.90} \right]$	$\left[ {0.15,0.90} \right]$	$\left[ {0.22,0.86} \right]$	$\left[ {0.31,0.74} \right]$
$y = 0.66$	$\left[ {0.20,0.85} \right]$	$\left[ {0.20,0.84} \right]$	$\left[ {0.25,0.80} \right]$	$\left[ {0.34,0.72} \right]$
$y = 1$	$\left[ {0.25,0.83} \right]$	$\left[ {0.31,0.80} \right]$	$\left[ {0.35,0.75} \right]$	$\left[ {0.35,0.71} \right]$
LMFs	$\tilde{Z}_{{\underline{1} }}$	$\tilde{Z}_{{\underline{2} }}$	$\tilde{Z}_{{\underline{3} }}$	$\tilde{Z}_{{\underline{4} }}$
$y = 0.33$	$\left[ {0.25,0.80} \right]$	$\left[ {0.26,0.80} \right]$	$\left[ {0.28,0.74} \right]$	$\left[ {0.39,0.66} \right]$
$y = 0.66$	$\left[ {0.30,0.77} \right]$	$\left[ {0.30,0.75} \right]$	$\left[ {0.35,0.70} \right]$	$\left[ {0.45,0.62} \right]$
$y = 1$	$\left[ {0.35,0.76} \right]$	$\left[ {0.59,0.70} \right]$	$\left[ {0.45,0.65} \right]$	$\left[ {0.46,0.59} \right]$
Defuzzified		0.5516

**Table 4:** Indicators weight.

Indicator	Weight	Relative weight
Mobile internet AP coverage	0.5501	0.0532
Virtualized resource pool stability	0.6147	0.0594
Maturity of smart city application system	0.6959	0.0673
Failure rate of software and hardware	0.7241	0.0700
Data theft and falsification	0.4512	0.0436
Development level of information industry	0.5278	0.0510
Security strategy and management	0.3097	0.0299
Security O&M management level	0.5516	0.0533
Perfection of special emergency plan	0.4313	0.0417
Incidence of accidents caused by responsibility and authority	0.4007	0.0387
Key personnel controlled	0.6567	0.0635
Practitioner intelligence level	0.7216	0.0698
Anti-virus software coverage	0.5749	0.0556
Access control and identity authentication	0.6811	0.0659
Data encryption and recovery	0.8005	0.0774
Perfection of information security standards	0.5419	0.0524
Information security legal binding	0.4775	0.0462
Public awareness of information security	0.6294	0.0609

**Figure 9:**
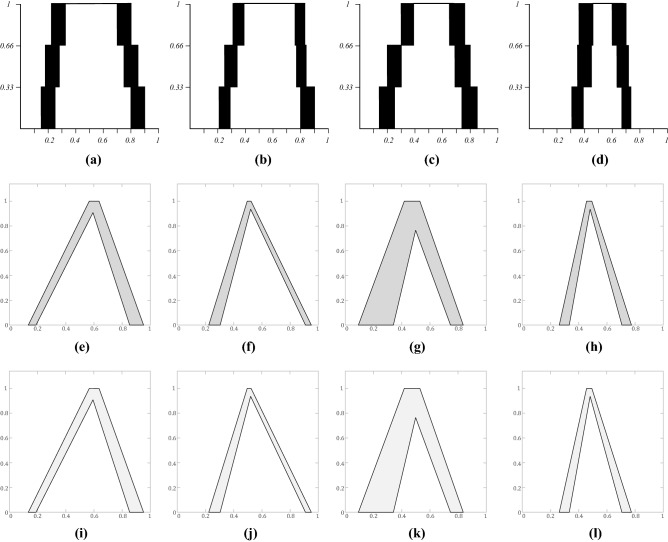
IT2 FSs, EIA and IA using corresponding crisp intervals for each expert **(a)** Expert A, **(b)** Expert B, **(c)** Expert C, **(d)** Expert D, **(e)** Expert A-EIA, **(f)** Expert B-EIA, **(g)** Expert C-EIA, **(h)** Expert D-EIA, **(i)** Expert A-IA, **(j)** Expert B-IA, **(k)** Expert C-IA, **(m)** Expert D-IA.

In order to verify the superiority of the IAA model, we conducted a comparison experiment of EIA and IA method use the same data of the 4 experts above according^44^ and^45^, results are shown directly as Fig. 9.

A direct comparison was showed above, it is apparent that the shape of the sets generated by three models have similarity, illustrates the effectiveness of the IAA method in evaluation. EIA and IA are two classic method of T2-fuzzistics methodology to obtain IT2 FS models that have already proved by many researches about their practical and validity^65–69^. Through above analysis, it can be known that IAA method models the intra-uncertainty in the primary membership, inter-uncertainty in the *FOU*, while EIA/IA method models the intra-uncertainty in the *FOU*, and do not capture the interval endpoints uncertainty. Next, the overall results demonstrate superiority of IAA when measuring different types of uncertainty (both inter and intra). EIA/IA combine both intra and inter-uncertainty in the triangular IT2 FS, different from the IAA using secondary membership to capture uncertainty across 4 experts, enable the capturing of both crisp and uncertain intervals, minimizing any loss of information and any assumptions.

Therefore, we summarize the different characteristics of the three methods and the superiority od IAA as:

- (1) if the decision come from a single source, which is, the intervals are crisp, IAA generates T1FS while IA produce IT2 FSs to measure intra-uncertainty based on single or repeated surveys;
- (2) if decisions come from multiple sources, which is, the intervals are crisp, IAA generates zGT2 FS to measure intra and inter uncertainty using primary and secondary membership while IA and EIA produce IT2 FS combining both types of uncertainty;
- (3) the IAA approach enables the capturing and modeling of uncertain intervals which is currently not directly possible with the IA/EIA approaches.

## Results and discussion

Based on the analysis results, the top 5 critical factors of smart city information security risk are: Data Encryption and Recovery (0.0774), Failure Rate of Software and Hardware (0.0700), Practitioner Intelligence Level (0.0698), Maturity of Smart City Application System (0.0673), Access Control and Identity Authentication (0.0659). From the results we deduct from the survey above, it can be seen that there are 2 index from the top 5 most important factors belong to the same category which is information security assurance (0.1989). And the rest of categories can be ranked by importance as information infrastructure (0.1799), information security personnel (0.1720), information technology (0.1647), information security environment (0.1594) and information management (0.1250). As the operations results show the rules and characters in the field of smart city information security, the policy orientation in the real world is also in agreement with it (we would give samples of those situation and cases in next paragraph), which prove that the methodology proposed in this paper can be used to analysis smart city information security during the government scientific decision-making process by giving the stakeholders a importance ranking reference, as they can use in relevant invest or policy-making programs.

In the context of the normalization of epidemic prevention and control, a large amount of data has been made publicly available to national research organizations in order to enhance epidemic traceability and prediction, leading to a significant increase in the difficulty of data encryption and recovery processing. Meanwhile, in the field of software and hardware, Huawei established the most stringent routing WIFI testing laboratory in Wuhan, 2019, gradually expanding its global market share through self-developed technology, and further enhancing China's global IT industry position. Further more, as the construction of smart cities continues, the construction of a new smart city puts forward higher requirements for the technological innovation and concept change of smart city practitioners, with a view to realizing the integration of smart city with financial technology, urban and rural planning, emergency decision-making and other fields. After Equifax and Alteryx data breaches, the need for authentication to protect privacy is increasing. The Chinese native open source server operating systems represented by Kylin focus on enhancing identity authentication, executing control mechanism and security audit^70^, but compared to the high level of security and reliability as it claimed, Kylin also faces problems like physical memory limits, unknown error occurred after resetting metadata^71^, etc.

In general, the development of China's smart city information security technology has achieved certain results, but still face many challenges. A large amount of foreign technologies and achievements have been applied in the core construction of China's smart city, bringing certain supply chain risks. In addition, with the world's urban development focusing on carbon emission and carbon neutrality, the construction and development of smart city have increased the strategic direction of reducing carbon emission, which puts forward new requirements for scientific and technological innovation and application. With the public attaching importance to the safety and sustainability of urban construction, the development of smart city in China should actively integrate social resources, strengthen technology R&D and promotion, truly realize the autonomy and controllability of core technology, and the refinement and intelligence of urban governance.

Based on the above analysis, we proposed the following strategy suggestions.

- (1) Strengthen the top-level design of information security in smart city. Government departments should conduct overall coordination from the top-level design, formulate all-round information security strategies, policies, plans and schemes, establish and improve the information security management mechanism of smart city, to avoid overlapping or blank areas of the functions of participating departments.
- (2) Build the smart city information security framework system. Combine the results from this paper above, optimizing access control is the focus and difficulty of managing information security risks in smart cities. In the application of smart city data, government should strengthen the security of the operating system, realize access control and hardware security through identity authentication technology and cloud storage security technology, to ensure the stability of the security system.
- (3) Improve the smart city information security evaluation mechanism. Security assessment can help the government and relevant departments effectively analyze system risks, master system security status, make scientific decisions, and improve the level of information security. Combined with the research of this paper, the government should fully consider the information uncertainty in decision-making, and comprehensively improve the information security evaluation mechanism of smart city from assets, threats, vulnerability and security measures.

## Conclusion

In this study, we discuss smart city information security risk prioritization problems using zGT2FSs and IAA method from the point of view of solving the problem of information loss in multi-criteria decision making. The results show that data encryption and recovery is the most critical factor affecting the smart city information security risk, and IAA method has apparently better ability to represent multidimensional uncertainty compared with EIA and IA. In the context of the normalization of COVID-19 prevention and control, it is very urgent to manage and protect a large number of data resources. As the operations results show the rules and characters in the field of smart city information security, the policy orientation in the real world is also in agreement with it, we suggest IAA method is very useful for capturing interval-based (survey) data and uncertainty information in fuzzy sets models by minimizing any assumptions or loss of information, which can supports crisp or uncertain intervals setting from multiple sources captured over different surveys. Compared with other MCDM methods such as VIKOR, TOPSIS, UAT etc., the IT2FSs & IAA method is also more realistic and easier to comprehend and implement.

However, there are still some deficiencies in this paper. Firstly, we only adopt 4 experts in this paper for case study, although there are advantages such as adequacy of the small number of decision makers and ease of application, limited to the computing complexity of high-class fuzzy logic system, the sample size is not big enough to a certain extent. Secondly, we have not proposed a practical case study from one or some smart cities in China as samples due to the general model data set limitation. In the future, we would aim to adopt reduct algorithms and machine learning to optimization computing process, enlarge the group number of experts, and we are also working on explore practical applications focusing on conducting a web- or mobile-app-based data collection exercise, which will expand the IAA method to access more representative data and evaluate the proposed approach in real-world contexts.

## Supplementary Information

Supplementary Information.

## References

[1] Zhan Y, Li S (2016). Financial development, technical innovation, and the construction of smart cities: An information development perspective. J. Finan. Econ..

[2] Li G, Li Y (2016). Construction of emergency decision-making intelligence system against the background of smart city. J. Library Sci. China.

[3] Yu W, Xu C (2016). Technological and political rationalities of smart city initiatives in China-An empirical analysis based on 147 cities. J. Public Manag..

[4] Lim H, Taeihagh A (2018). Autonomous vehicles for smart and sustainable Cities: An in-depth exploration of privacy and cybersecurity implications. Energies.

[5] Wang J, Li C, Xiong Z (2014). Survey of data-centric smart city. J. Comput. Res. Dev..

[6] Tang S, Zhang Y, Shan Z (2020). Development status, situation and policy suggestions of new smart city in China. E-Government.

[7] Hadi H, Brian H, Fazel A (2019). A survey on cybersecurity, data privacy, and policy issues in cyber-physical system deployments in smart cities. Sustain. Cities Soc..

[8] Xu H (2020). Developing smart cities based on “digital twin”. Frontiers.

[9] Yin L, Zhang C (2019). Summary of theoretical research and practical progress of smart city in China. E-Government.

[10] Harrison C, Eckman B, Hamilton R (2010). Foundations for smarter cities. IBM J. Res. Dev..

[11] Barlow M, Lévy-Bencheton C (2018). Smart City, Smart Future: Showcasing Tomorrow.

[12] Gassmann O, Jonas B, Maximilian P (2019). Smart Cities: Introducing Digital Innovation to Cities.

[13] Anthopoulos LG (2017). Understanding Smart Cities: A Tool for Smart Government or an Industrial Trick?.

[14] Zhuhadar L, Thrasher E, Marklin S (2017). The next wave of innovation-review of smart cities intelligent operation systems. Comput. Hum. Behav..

[15] Mattoni B, Gugliermetti F, Bisegna F (2015). A multilevel method to assess and design the renovation and integration of smart cities. Sustain. Cities Soc..

[16] Xiang S, Zou K, Jiang Z (2016). Risk prediction of smart city information security based on random forest. Chinese J. Manag. Sci..

[17] Hou, L. *Information Security Risks of Smart Cities Influencing Factors. Master Thesis*. (Xiangtan University, 2020).

[18] Mao Z, Mei H, Xiao Y (2020). Risk assessment of smart city information security based on Bayesian network. J. Mod. Inf..

[19] Felipe, S., & Carlos, A. *Smart City Security Issues: Depicting Information Security Issues in the Role of a Urban Environment*. in *2014 IEEE/ACM 7th International Conference on Utility and Cloud Computing**, **London* (2014).

[20] Moch N, Wereda W (2020). Smart security in the smart city. Sustainability..

[21] Wang Y, Li L, Yu H (2021). Construction of risk and security system for information security of smart city. Video Eng..

[22] Sun J, Pei L, Qiu P (2016). The challenge and success experience of smart city initiatives-based on literature review and case analysis. Library Inf..

[23] Yunbo L, Anshen L (2013). Analysis of the challenges and solutions of building a smart city. Int. Conf. Constr. Real Estate Manag..

[24] Zou K, Xiang S, Zhang Z (2016). Model construction and empirical study on smart city information security risk assessment. Library Inf. Serv..

[25] Huang J, Dou L, Fang H (2015). Distributed backstepping-based adaptive fuzzy control of multiple high-order nonlinear dynamics. Nonlinear Dyn..

[26] Chen CLP, Ren C, Du T (2016). Fuzzy observed-based adaptive consensus tracking control for second-order multiagent systems with heterogeneous nonlinear dynamics. IEEE Trans. Fuzzy Syst..

[27] Oscar C, Leticia A, Juan R (2016). A comparative study of type-1 fuzzy logic systems, interval type-2 fuzzy logic systems and generalized type-2 fuzzy logic systems in control problems. Inf. Sci..

[28] Bustince H (2016). A historical account of types of fuzzy sets and their relationships. IEEE Trans. Fuzzy Syst..

[29] Rodríguez RM, Martínez L, Torra V (2014). Hesitant fuzzy sets: State of the art and future directions. Int. J. Intell. Syst..

[30] Wagner C, Hagras H (2010). Toward general type-2 fuzzy logic systems based on zSlices. IEEE Trans. Fuzzy Syst..

[31] Benzmüller C (2011). Combining and automating classical and non-classical logics in classical higher-order logics. Ann. Math. Artif. Intell..

[32] Rohrlich F (2011). Why physics needs mathematics. Gen. Relativ. Gravity.

[33] Dellunde P, García-Cerdaña À, Noguera C (2016). Löwenheim-Skolem theorems for non-classical first-order algebraizable logics. Logic J. IGPL.

[34] Zadeh L (1965). Fuzzy sets. Inf. Control.

[35] Zadeh L (1975). The concept of a linguistic variable and its application to approximate reasoning—I. Inf. Sci..

[36] Patricia M, Oscar C (2014). A review on type-2 fuzzy logic applications in clustering, classification and pattern recognition. Appl. Soft Comput..

[37] Wu D, Mendel JM (2009). A comparative study of ranking methods, similarity measures and uncertainty measures for interval type-2 fuzzy sets. Inf. Sci..

[38] Karnik NN, Mendel JM (2001). Operations on type-2 fuzzy sets. Fuzzy Sets Syst..

[39] Sarah G, Francisco C, Simon C (2009). The collapsing method of defuzzification for discretised interval type-2 fuzzy sets. Inf. Sci..

[40] Mendel JM (2014). General type-2 fuzzy logic systems made simple: A tutorial. IEEE Trans. Fuzzy Syst..

[41] Mendel JM, John RIB (2002). Type-2 fuzzy sets made simple. IEEE Trans. Fuzzy Syst..

[42] Coupland S, John R (2007). Geometric type-1 and type-2 fuzzy logic systems. IEEE Trans. Fuzzy Syst..

[43] Mendel JM, Hagras H, Tan W (2014). Introduction to Type-2 Fuzzy Logic Control: Theory and Application.

[44] Liu F, Mendel J (2008). Encoding words into interval type-2 fuzzy sets using an interval approach. IEEE Trans. Fuzzy Syst..

[45] Coupland S, Mendel J, Wu D (2010). Enhanced interval approach for encoding words into interval type-2 fuzzy sets and convergence of the word FOUs. Proc. IEEE Int. Conf. Fuzzy Syst..

[46] Wu D, Mendel J, Coupland S (2012). Enhanced interval approach for encoding words into interval type-2 fuzzy sets and its convergence analysis. IEEE Trans. Fuzzy Syst..

[47] Dongrui WU, Zhigang ZENG, Hong MO (2020). Interval type-2 fuzzy sets and systems: Overview and outlook. ACTA Autom. Sin..

[48] Wagner C, Miller S, Garibaldi JM (2014). From interval-valued data to general type-2 fuzzy sets. IEEE Trans. Fuzzy Syst..

[49] Chao F, Zhou D, Lin C (2020). Type-2 fuzzy hybrid controller network for robotic systems. IEEE Trans. Cybern..

[50] McCulloch J, Wagner C (2020). On the choice of similarity measures for type-2 fuzzy sets. Inf. Sci..

[51] Wu D, Mendel JM (2019). Similarity measures for closed general type-2 fuzzy sets: Overview, comparisons, and a geometric approach. IEEE Trans. Fuzzy Syst..

[52] Havens TC, Anderson DT, Wagner C (2015). Data-informed fuzzy measures for fuzzy integration of intervals and fuzzy numbers. IEEE Trans. Fuzzy Syst..

[53] X. Jinping. *Build a Moderately Prosperous Society in an All Round Way and Win the Great Victory of Socialism with Chinese Characteristics in the New Era*. in *Report at the 19th National Congress of the Communist Party of China*. http://cpc.people.com.cn/n1/2017/1028/c64094-29613660.html.

[54] Li X, Li H, Sun B (2018). Assessing information security risk for an evolving smart city based on fuzzy and grey FMEA. J. Intell. Fuzzy Syst..

[55] Andrea L, Giacomo L (2014). RFID-plants in the smart city: Applications and outlook for urban green management. Urban For. Urban Green..

[56] Haeckel E (1866). Generelle Morphologie Der Organismen.

[57] Shihai T, Jiayu Z, Meiqi S (2020). Research on the derivation of internet public opinion information ecological community based on improved SIR. Inf. Sci..

[58] Jayavardhana G, Rajkumar B, Slaven M (2013). Internet of things (IoT): A vision, architectural elements, and future directions. Futur. Gener. Comput. Syst..

[59] Schmidt KA, Sasha D, Jan A (2010). The ecology of information: An overview on the ecological significance of making informed decisions. Oikos.

[60] Nazi KM (2013). The personal health record paradox: Health care professionals' perspectives and the information ecology of personal health record systems in organizational and clinical settings. J. Med. Internet Res..

[61] Finin T, Joshi A, Kolari P (2008). The information ecology of social media and online communities. AI Mag..

[62] Dou Y (2020). Research on construction of “3×3” emergency intelligence system from the perspective of information ecology. Library Inf. Serv..

[63] Wei F (2021). Study on theory, method and optimization countermeasures of network information ecological environment evaluation. Inf. Sci..

[64] Bekkers VJJM, Homburg V (2005). The Information Ecology of e-Government: e-Government as Institutional and Technological Innovation in Public Administration.

[65] Jennison C, Turnbull BW (1989). Interim analyses: The repeated confidence interval approach. J. R. Stat. Soc.: Ser. B (Methodol.).

[66] King RP, Robison LJ (1981). An interval approach to measuring decision maker preferences. Am. J. Agric. Econ..

[67] Cocks K, Torgerson DJ (2013). Sample size calculations for pilot randomized trials: A confidence interval approach. J. Clin. Epidemiol..

[68] Coupland S, Mendel JM, Wu D (2010). Enhanced interval approach for encoding words into interval type-2 fuzzy sets and convergence of the word fous. Int. Conf. Fuzzy Syst. IEEE..

[69] Mendel JM (2016). A comparison of three approaches for estimating (synthesizing) an interval type-2 fuzzy set model of a linguistic term for computing with words. Granular Comput..

[70] Lv P, Zhao R, Wang C (2021). Design and implementation of SRIO routing based on NeoKylin system. Radio Eng..

[71] Liu K, Gu J, Du Y (2019). Development of PXI instrument software based on QT and NeoKylin operating system. Comput. Meas. Control.

