# Supplementary material for: The assessment of smart city information security risk in China based on zGT2FSs and IAA method
Source: Sci Rep. 2022 Feb 28;12:3281. doi: 10.1038/s41598-022-07197-1 (PMC8885652; doi:10.1038/s41598-022-07197-1)
Supplement: Supplementary file 1 — Supplementary Information. [file 41598_2022_7197_MOESM1_ESM.docx]

**Appendix I**

1. **Classify indicators according information ecology theory**

Information ecology is an organic whole composed of information people, communities, organizations, information behavior, values and information technology in a certain environment. Information ecology theory is the theoretical basis of information ecosystem and its application. The core idea is to emphasize the relationship between people and environment, advocate reasonable differentiation of information ecological niche, normalize the function of information ecosystem and achieve some kind of balance (Figure I). Under this theory, many papers gave variance classification of information system, which can be summarized to information producer, information server, information consumer, information supervisor and information environment.

**Figure I. Information Ecology Schematic**

We combine the theory proposed by Haeckel, deduct the smart city information security into 6 aspects including infrastructure, technology, management, personnel, assurance and environment.

1. **Literature retrieval**

The following retrieval strategy was adopted to explore smart city information security studies in SCOPE from 2000 to 2020: TITLE-ABS-KEY (“smart city” OR “smart cities” OR “smart city security” OR “smart city security evaluation” OR “smart cities security” OR “smart cities security evaluation” OR “smart sustainable cities” OR “smart city information model” OR “intelligent cities”OR “smarter cities”OR “smart city information security risk” OR “information security risk evaluation”) AND DOCTYPE (ar OR re) AND SUBJAREA (ener OR engi OR envi OR deci OR econ OR soci) AND PUBYEAR AFT 2001 AND PUBYEAR BEF 2020 AND (LIMIT-TO (LANGUAGE, “English”)) AND (LIMIT -TO(SRCTYPE, “j”)).

Based on above search rules, we find the indicators have high occurrence frequency (**Table I**), which indicate those indicator possess academic scientific.

**Table I. Indicators from literature retrieval**

| First-level Indicators | Second-level Indicators | Ref |
| --- | --- | --- |
| Information infrastructure | Localization rate of core equipment | [^[[1]](#endnote-0)^] |
|  | IoT infrastructure coverage | [^[[2]](#endnote-1)^] |
|  | Mobile Internet AP Coverage | [^[[3]](#endnote-2)^] |
|  | Virtualized Resource Pool Stability | [^[[4]](#endnote-3)^] |
|  | Maturity of Smart City Application System | [^[[5]](#endnote-4)^] |
|  | Perfection of Security Review System | [^[[6]](#endnote-5)^] |
|  | Vulnerability Scanning Security Index | [2] |
|  | Third Party Vulnerabilities Security Index | [^[[7]](#endnote-6)^] |
|  | Vulnerability Attack Security Index | [^[[8]](#endnote-7)^] |
| Information Technology | Failure Rate of Software and Hardware | [^[[9]](#endnote-8)^] |
|  | Physical Environment Impact | [^[[10]](#endnote-9)^] |
|  | Data Theft | [^[[11]](#endnote-10)^] |
|  | Data Falsification | [^[[12]](#endnote-11)^] |
|  | Number of Computer Malware | [2] |
|  | Weak password camera ratio | [^[[13]](#endnote-12)^] |
|  | Number of Information Security Technology Patents | [^[[14]](#endnote-13)^] |
|  | Development Level of Information Industry | [^[[15]](#endnote-14)^] |
| Information Management | Security Strategy and Management System Perfection | [^[[16]](#endnote-15)^] |
|  | Security O&M Management Level | [^[[17]](#endnote-16)^] |
|  | Perfection of Special Emergency Plan | [^[[18]](#endnote-17)^] |
|  | Personnel Changes in Key Departments | [^[[19]](#endnote-18)^] |
| Information Security Personnel | Number of Registered Information Security Professionals | [^[[20]](#endnote-19)^] |
|  | Incidence of Accidents Caused by Responsibility and Authority | [^[[21]](#endnote-20)^] |
|  | Misoperation | [^[[22]](#endnote-21)^] |
|  | Key Personnel Controlled | [^[[23]](#endnote-22)^] |
|  | Professional Ethics | [^[[24]](#endnote-23)^] |
|  | Practitioner Intelligence Level | [^[[25]](#endnote-24)^] |
| Information Security Assurance | Intrusion Prevention Detection | [^[[26]](#endnote-25)^] |
|  | Applicability of Emergency Plan | [^[[27]](#endnote-26)^] |
|  | Safety Educating and Training | [^[[28]](#endnote-27)^] |
|  | Management and Supervision System | [25] |
|  | Anti-virus Software Coverage | [21] |
|  | Patch Upgrade Rate | [3] |
|  | Access Control | [4] |
|  | Identity Authentication | [28] |
|  | Data Encryption and Auditing | [^[[29]](#endnote-28)^] |
|  | Data Backup and Restore | [^[[30]](#endnote-29)^] |
| Information Security Environment | Perfection of Information Security Standards | [18] |
|  | Information Security Legal Binding | [^[[31]](#endnote-30)^] |
|  | Public Awareness of Information Security | [10] |
|  | Aggressive Service Providers | [1] |
|  | Competitive Pressure | [13] |

1. **Coefficient of variation method**

We conduct expert interview, details and results are summarized below as Table II and Table III.

**Table II. Experts Resource**

| **Category** | **Number** |
| --- | --- |
| Government Department | 3 |
| University/Institute | 4 |
| Smart City Application Development | 2 |
| Data Service | 4 |
| Circuits/Grids | 2 |
| Mobile Internet | 2 |
| Risk assessment | 1 |

**Table.III Variation Coefficients**

| **Indicators** | **Average** | **Coefficient of Variation** | **Indicators** | **Average** | **Coefficient of Variation** |
| --- | --- | --- | --- | --- | --- |
| Localization Rate of Core Equipment | 3.444 | 0.3898 | Number of Registered Information Security Professionals | 2.556 | 0.2680 |
| IoT Infrastructure Coverage | 3.889 | 0.1895 | Incidence of Accidents Caused by Responsibility and Authority | 3.889 | 0.2250 |
| Mobile Internet AP Coverage | 4.333 | 0.1538 | Misoperation | 3.556 | 0.2339 |
| Virtualized Resource Pool Stability | 4.111 | 0.2676 | Key Personnel Controlled | 3.667 | 0.1818 |
| Maturity of Smart City Application System | 3.667 | 0.2227 | Professional Ethics | 3.889 | 0.1457 |
| Perfection of Security Review System | 4.556 | 0.1091 | Practitioner Intelligence Level | 3.333 | 0.2000 |
| Vulnerability Scanning Security Index | 4.000 | 0.2041 | Intrusion Prevention Detection | 4.333 | 0.1088 |
| Third Party Vulnerabilities Security Index | 3.778 | 0.2080 | Applicability of Emergency Plan | 4.222 | 0.1861 |
| Vulnerability Attack Security Index | 4.333 | 0.1088 | Safety Educating and Training | 3.444 | 0.1443 |
| Failure Rate of Software and Hardware | 3.889 | 0.1895 | Management and Supervision System | 3.778 | 0.1664 |
| Physical Environment Impact | 3.000 | 0.2722 | Anti-virus Software Coverage | 4.111 | 0.2128 |
| Data Theft | 4.667 | 0.1429 | Patch Upgrade Rate | 2.667 | 0.3062 |
| Data Falsification | 4.778 | 0.0870 | Access Control | 3.889 | 0.1895 |
| Number of Computer Malware | 4.000 | 0.2357 | Identity Authentication | 4.111 | 0.2128 |
| Weak password camera ratio | 3.000 | 0.0000 | Data Encryption and Auditing | 4.778 | 0.1316 |
| Number of Information Security Technology Patents | 3.111 | 0.3536 | Data Backup and Restore | 2.778 | 0.0870 |
| Development Level of Information Industry | 3.778 | 0.1664 | Perfection of Information Security Standards | 3.889 | 0.1457 |
| Security Strategy and Management System Perfection | 4.000 | 0.1667 | Information Security Legal Binding | 4.111 | 0.2911 |
| Security O&M Management Level | 3.889 | 0.1457 | Public Awareness of Information Security | 3.889 | 0.1457 |
| Perfection of Special Emergency Plan | 3.778 | 0.2080 | Aggressive Service Providers | 2.333 | 0.4518 |
| Personnel Changes in Key Departments | 2.111 | 0.4144 | Competitive Pressure | 2.667 | 0.1768 |

In general, when the average of experts' scores on indicators is greater than 3 and the coefficient of variation is less than 0.25, the indicators can be retained; the coefficient of variation represents the amount of information contained in the indicators, if the degree of variation of an indicator is 0, it means that all the evaluation objects have equal observations on the indicator, and the indicator has no value for evaluation.

After the above steps, we delete 24 indicators according to experts opinion, do sort and optimize, finally give the indicator system as **Figure 5** in **3.2**.

**Appendix II**

1. **Uncertain Intervals to Interval Type-2 Fuzzy Sets**

$+y_{2}/\left((\left[0.25,0.90\right]\cap\left[0.20,0.85\right])\cup(\left[0.25,0.90\right]\cap\left[0.15,0.80\right])\cup(\left[0.20,0.85\right]\cap\left[0.15,0.80\right])\right)$ $+y_{3}/\left(\left[0.25,0.90\right]\cap\left[0.20,0.85\right]\cap\left[0.15,0.80\right]\right)$

$$=\left(y_{1}/\left[0.15,0.90\right]+y_{2}/\left[0.20,0.85\right]+y_{3}/\left[0.25,0.80\right]\right)$$

$$\underline{\mu}\left(\tilde{A}\right)()=y_{1}/\left(\left[0.35,0.80\right]\cup\left[0.30,0.75\right]\cup\left[0.25,0.70\right]\right)$$

$$+y_{2}/\left((\left[0.35,0.80\right]\cap\left[0.30,0.75\right])\cup(\left[0.35,0.80\right]\cap\left[0.25,0.70\right])\cup(\left[0.30,0.75\right]\cap\left[0.25,0.70\right])\right)$$

$$+y_{3}/\left(\left[0.35,0.80\right]\cap\left[0.30,0.75\right]\cap\left[0.25,0.70\right]\right)$$

$$=\left(y_{1}/\left[0.25,0.80\right]+y_{2}/\left[0.30,0.75\right]+y_{3}/\left[0.35,0.70\right]\right)$$

**(2) Interval Type-2 Fuzzy Sets to General Type-2 Fuzzy Sets**

$$+y_{2}/\left(\left[0.20,0.85\right]\cup\left[0.25,0.84\right]\cup\left[0.20,0.80\right]\cup\left[0.34,0.72\right]\right)$$

$$+y_{3}/\left(\left[0.25,0.80\right]\cup\left[0.31,0.83\right]\cup\left[0.35,0.75\right]\cup\left[0.35,0.71\right]\right)\text{）}$$

$$=0.25/\left(\left(0.33/\left[0.14,0.90\right]\right)+\left(0.66/\left[0.20,0.85\right]\right)+\left(1/\left[0.25,0.83\right]\right)\right)$$

$$\tilde{Z}_{\underline{1}}=0.25/(y_{1}/\left(\left[0.25,0.80\right]\cup\left[0.28,0.80\right]\cup\left[0.26,0.74\right]\cup\left[0.39,0.66\right]\right)$$

$$+y_{2}/\left(\left[0.30,0.75\right]\cup\left[0.35,0.77\right]\cup\left[0.30,0.70\right]\cup\left[0.45,0.62\right]\right)$$

$$+y_{3}/\left(\left[0.35,0.70\right]\cup\left[0.39,0.76\right]\cup\left[0.45,0.65\right]\cup\left[0.46,0.59\right]\right)\text{）}$$

$$=0.25/\left(\left(0.33/\left[0.25,0.80\right]\right)+\left(0.66/\left[0.30,0.77\right]\right)+\left(1/\left[0.35,0.76\right]\right)\right)$$

$\cup\left(\left[0.22,0.90\right]\cap\left[0.14,0.86\right]\right)\cup\left(\left[0.22,0.90\right]\cap\left[0.31,0.74\right]\right)\cup\left(\left[0.14,0.86\right]\cap\left[0.31,0.74\right]\right)$

$$+y_{2}/\left(\left[0.20,0.85\right]\cap\left[0.25,0.84\right]\right)\cup\left(\left[0.20,0.85\right]\cap\left[0.20,0.80\right]\right)\cup\left(\left[0.20,0.85\right]\cap\left[0.34,0.72\right]\right)$$

$$\cup\left(\left[0.25,0.84\right]\cap\left[0.20,0.80\right]\right)\cup\left(\left[0.25,0.84\right]\cap\left[0.34,0.72\right]\right)\cup\left(\left[0.20,0.80\right]\cap\left[0.34,0.72\right]\right)$$

$$+y_{3}/\left(\left[0.25,0.80\right]\cap\left[0.31,0.83\right]\right)\cup\left(\left[0.25,0.80\right]\cap\left[0.35,0.75\right]\right)\cap\left(\left[0.25,0.80\right]\cap\left[0.35,0.71\right]\right)\text{）}$$

$$\cup\left(\left[0.31,0.83\right]\cap\left[0.35,0.75\right]\right)\cup\left(\left[0.31,0.83\right]\cap\left[0.35,0.71\right]\right)\cap\left(\left[0.35,0.75\right]\cap\left[0.35,0.71\right]\right)\text{）}$$

$$=0.5/\left(\left(0.33/\left[0.15,0.90\right]\right)+\left(0.66/\left[0.20,0.84\right]\right)+\left(1/\left[0.31,0.80\right]\right)\right)$$

$\tilde{Z}_{\underline{2}}=0.5/(y_{1}/\left(\left[0.25,0.80\right]\cap\left[0.28,0.80\right]\right)\cup\left(\left[0.25,0.80\right]\cap\left[0.26,0.74\right]\right)\cup\left(\left[0.25,0.80\right]\cap\left(\left[0.39,0.66\right]\right)\right)$ $\cup\left(\left[0.28,0.80\right]\cap\left[0.26,0.74\right]\right)\cup\left(\left[0.28,0.80\right]\cap\left[0.39,0.66\right]\right)\cap\left(\left[0.26,0.74\right]\cap\left[0.39,0.66\right]\right)$

$$+y_{2}/\left(\left[0.30,0.75\right]\cap\left[0.35,0.77\right]\right)\cup\left(\left[0.30\text{，}0.75\right]\cap\left[0.30,0.70\right]\right)\cap\left(\left[0.30,0.75\right]\cap\left[0.45,0.62\right]\right)$$

$$\cup\left(\left[0.35,0.77\right]\cap\left[0.30,0.70\right]\right)\cup\left(\left[0.35,0.77\right]\cap\left[0.45,0.62\right]\right)\cap\left(\left[0.30,0.70\right]\cap\left[0.45,0.62\right]\right)$$

$$+y_{3}/\left(\left[0.35,0.70\right]\cap\left[0.39,0.76\right]\right)\cup\left(\left[0.35,0.70\right]\cap\left[0.45,0.65\right]\right)\cap\left(\left[0.35,0.70\right]\cap\left[0.46,0.59\right]\right)$$

$$\cup\left(\left[0.39,0.76\right]\cap\left[0.45,0.65\right]\right)\cup\left(\left[0.39,0.76\right]\cap\left[0.46,0.59\right]\right)\cap\left(\left[0.45,0.65\right]\cap\left[0.46,0.59\right]\right)\text{）}$$

$$=0.5/\left(\left(0.33/\left[0.26,0.80\right]\right)+\left(0.66/\left[0.30,0.75\right]\right)+\left(1/\left[0.39,0.70\right]\right)\right)$$

$$\cup\left(\left[0.15,0.90\right]\cap\left[0.14,0.86\right]\cap\left[0.31,0.74\right]\right)\cup\left(\left[0.22,0.90\right]\cap\left[0.14,0.86\right]\cap\left[0.31,0.74\right]\right)$$

$$+y_{2}/\left(\left[0.20,0.85\right]\cap\left[0.25,0.84\right]\cap\left[0.20,0.80\right]\right)\cup\left(\left[0.20,0.85\right]\cap\left[0.25,0.84\right]\cap\left[0.34,0.72\right]\right)$$

$$\cup\left(\left[0.20,0.85\right]\cap\left[0.20,0.80\right]\cap\left[0.34,0.72\right]\right)\cup\left(\left[0.25,0.84\right]\cap\left[0.20,0.80\right]\cap\left[0.34,0.72\right]\right)$$

$$+y_{3}/\left(\left[0.25,0.80\right]\cap\left[0.31,0.83\right]\cap\left[0.35,0.75\right]\right)\cup\left(\left[0.25,0.80\right]\cap\left[0.31,0.83\right]\cap\left[0.35,0.71\right]\right)$$

$$\cup\left(\left[0.31,0.83\right]\cap\left[0.35,0.75\right]\cap\left[0.35,0.71\right]\right)\cup\left(\left[0.25,0.80\right]\cap\left[0.35,0.75\right]\cap\left[0.35,0.71\right]\right)\text{）}$$

$$=0.75/\left(\left(0.33/\left[0.22,0.86\right]\right)+\left(0.66/\left[0.25,0.80\right]\right)+\left(1/\left[0.35,0.75\right]\right)\right)$$

$\tilde{Z}_{\underline{3}}=0.75/(y_{1}/\left(\left[0.25\text{，}0.80\right]\cap\left[0.28,0.80\right]\cap\left[0.26,0.74\right]\right)\cup\left(\left[0.25,0.80\right]\cap\left[0.28,0.80\right]\cap\left[0.39,0.66\right]\right)$ $\cup\left(\left[0.28,0.80\right]\cap\left[0.26,0.74\right]\cap\left[0.39,0.66\right]\right)\cup\left(\left[0.25,0.80\right]\cap\left[0.26,0.74\right]\cap\left[0.39,0.66\right]\right)$

$$+y_{2}/\left(\left[0.30,0.75\right]\cap\left[0.35,0.77\right]\cap\left[0.30,0.70\right]\right)\cup\left(\left[0.30,0.75\right]\cap\left[0.30,0.70\right]\cap\left[0.45,0.62\right]\right)$$

$$\cup\left(\left[0.35,0.75\right]\cap\left[0.35,0.70\right]\cap\left[0.45,0.62\right]\right)\cup\left(\left[0.35,0.77\right]\cap\left[0.30,0.70\right]\cap\left[0.45,0.62\right]\right)$$

$$+y_{3}/\left(\left[0.35,0.70\right]\cap\left[0.39,0.76\right]\cap\left[0.45\text{，}0.65\right]\right)\cup\left(\left[0.35,0.70\right]\cap\left[0.39,0.76\right]\cap\left[0.46,0.59\right]\right)$$

$$\cup\left(\left[0.35,0.70\right]\cap\left[0.45,0.65\right]\cap\left[0.46,0.59\right]\right)\cup\left(\left[0.39,0.76\right]\cap\left[0.45,0.65\right]\cap\left[0.46,0.59\right]\right)\text{）}$$

$$=0.75/\left(\left(0.33/\left[0.28,0.74\right]\right)+\left(0.66/\left[0.35,0.70\right]\right)+\left(1/\left[0.45,0.65\right]\right)\right)$$

$$+y_{2}/\left(\left[0.20,0.85\right]\cap\left[0.25,0.84\right]\cap\left[0.20,0.80\right]\cap\left[0.34,0.72\right]\right)$$

$$+y_{3}/\left(\left[0.25,0.80\right]\cap\left[0.31,0.83\right]\cap\left[0.35,0.75\right]\cap\left[0.35,0.71\right]\right)\text{）}$$

$$=1/\left(\left(0.33/\left[0.31\text{，}0.74\right]\right)+\left(0.66/\left[0.34,0.72\right]\right)+\left(1/\left[0.35,0.71\right]\right)\right)$$

$$\tilde{Z}_{\underline{4}}=1/(y_{1}/\left(\left[0.25,0.80\right]\cap\left[0.28,0.80\right]\cap\left[0.26,0.74\right]\cap\left[0.39,0.66\right]\right)$$

$$+y_{2}/\left(\left[0.30,0.75\right]\cap\left[0.35,0.77\right]\cap\left[0.30,0.70\right]\cap\left[0.45,0.62\right]\right)$$

$$+y_{3}/\left(\left[0.35,0.70\right]\cap\left[0.39,0.76\right]\cap\left[0.45,0.65\right]\cap\left[0.46,0.59\right]\right)\text{）}$$

$$=1/\left(\left(0.33/\left[0.39,0.66\right]\right)+\left(0.66/\left[0.45,0.62\right]\right)+\left(1/\left[0.46,0.59\right]\right)\right)$$

**References**

1. [] Cocks K, Torgerson D J. Sample size calculations for pilot randomized trials: a confidence interval approach, *Journal of clinical epidemiology*, **2013**, 66(2): 197-201. [↑](#endnote-ref-0)
2. [] Hong S, Park S, Park L W, et al. An analysis of security systems for electronic information for establishing secure internet of things environments: Focusing on research trends in the security field in South Korea, *Future Generation Computer Systems*, **2018**, 82: 769-782. [↑](#endnote-ref-1)
3. [] Park W, Na O, Chang H. An exploratory research on advanced smart media security design for sustainable intelligence information system, *Multimedia Tools and Applications*, **2016**, 75(11):6059-6070. [↑](#endnote-ref-2)
4. [] Lundgren Bjorn, Moller Niklas. Defining Information Security, *Science and Engineering Ethics*, **2019**, 25(2):419- 441. [↑](#endnote-ref-3)
5. [5] Li D, Yao Y, Shao Z. Big Data in Smart City, *Geomatics and Information Science of Wuhan University*, **2014**, 39(06): 631-640. [↑](#endnote-ref-4)
6. [] Webb J, Ahmad A, Maynard S B, et al. A situation awareness model for information security risk management, *Computers & Security*, **2014**, 44: 1-15. [↑](#endnote-ref-5)
7. [] Chang K, Wang C. Information systems resources and information security, *Information Systems Frontiers*, **2011**, 13(4): 579-593 [↑](#endnote-ref-6)
8. [] Von Solms R, Van Niekerk J. From information security to cyber security, *Computers & Security*, **2013**, 38: 97-102. [↑](#endnote-ref-7)
9. [] Dai Z, Wang Z, Jiao Y. Dynamic reliability assessment of protection system based on dynamic fault tree and Monte Carlo simulation, *Proceedings of the CSEE*, **2011**, 31(19): 105-113 [↑](#endnote-ref-8)
10. [] Ma J, Hu M, Lian M. Design of Smart City Information Ecological Chain Based on Multi-subject Collaboration, *Information Science*, **2016**, 34(12):70-74, 81. [↑](#endnote-ref-9)
11. [] Fu Y, Wu X, Ye Q, et al. An Approach for Information Systems Security Risk Assessment on Fuzzy Set and Entropy-Weight, *Acta Electronica Sinica*, **2010**, 38(07):1489-1494. [↑](#endnote-ref-10)
12. [] Jin R, He X, Dai H. On the security-privacy tradeoff in collaborative security: A quantitative information flow game perspective, *IEEE Transactions on Information Forensics and Security*, **2019**, 14(12): 3273-3286. [↑](#endnote-ref-11)
13. [] Qiu Y, Li S. Security Threat Analysis and Solutions for the Development and Application of Artificial Intelligence, *Netinfo Security*, **2018**, 09:35-41. [↑](#endnote-ref-12)
14. [] Sun G, Wei Y, Luo D. Research on Intellectual Property Competition Situation of Chinese and Foreign Information Security Enterprises, *Electronics Intellectual Property*, **2005**, 03:27-32. [↑](#endnote-ref-13)
15. [] Wu Y, Sun R, Wu Y. Smart City Development in Taiwan: From the Perspective of the Information Security Policy, *Sustainability*, **2020**, 12(7). [↑](#endnote-ref-14)
16. [] Dong K, Lin R, Yin X, Xie Z. How does overconfidence affect information security investment and information security performance?, *Enterprise Information Systems*, **2021**, 15(4): 474-491. [↑](#endnote-ref-15)
17. [] Albrechtsen E, Hovden J. The information security digital divide between information security managers and users. *Computers & Security*, **2009**, 28(6): 476-490 [↑](#endnote-ref-16)
18. [] Lin C, Kunnathur A S, Li L. The cultural foundation of information security behavior: Developing a cultural fit framework for information security behavior control. *Journal of Database Management (JDM)*, **2020**, 31(2): 21-41 [↑](#endnote-ref-17)
19. [] Yu H. The opportunity,challenges and solutions of the management of government data in the Age of Big Data. *Chinese Public Administration*, **2015**, 30:127-130. [↑](#endnote-ref-18)
20. [] Siponen M, Willison R. Information security management standards: Problems and solutions. *Information & management*, **2009**, 46(5): 267-270. [↑](#endnote-ref-19)
21. [] Siponen M, Mahmood M A, Pahnila S. Employees’ adherence to information security policies: An exploratory field study. *Information & management*, **2014**, 51(2): 217-224. [↑](#endnote-ref-20)
22. [] Du Y, Du J, Chen J. System of Measuring and Appraising the Quality of Practitioners of Information Security in E-commerce. *Systems Engineering-Theory & Practice*, **2010**, 30(10):1870-1876. [↑](#endnote-ref-21)
23. [] Yang S, Zhu J. Risk Management Model and Preventive Measures of Information Security Based on Internal Threat. *Modernization of Management*, **2013**, 02:47-49. [↑](#endnote-ref-22)
24. [] Posey C, Roberts T L, Lowry P B, et al. Bridging the divide: A qualitative comparison of information security thought patterns between information security professionals and ordinary organizational insiders. *Information & Management*, **2014**, 51(5): 551-567. [↑](#endnote-ref-23)
25. [] Kritzinger E, Smith E. Information security management: An information security retrieval and awareness model for industry. *Computers & Security*, **2008**, 27(5-6): 224-231. [↑](#endnote-ref-24)
26. [] Govender S G, Kritzinger E, Loock M. A framework and tool for the assessment of information security risk, the reduction of information security cost and the sustainability of information security culture. *Personal and Ubiquitous Computing*, **2021**: 1-14. [↑](#endnote-ref-25)
27. [] Cholez H, Girard F. Maturity assessment and process improvement for information security management in small and medium enterprises. *Journal of Software: Evolution and Process*, **2014**, 26(5): 496-503. [↑](#endnote-ref-26)
28. [] Vural Y, Sagiroglu Ş. A review on enterprise information security and standards. 2008. [↑](#endnote-ref-27)
29. [] Bloch M, Barros J, Rodrigues M R D, et al. Wireless information-theoretic security. *IEEE Transactions on Information Theory*, **2008**, 54(6): 2515-2534 [↑](#endnote-ref-28)
30. [] Yuan Y, Ding J, Lu Y, et al. Automatic Management Information System for Protective Relaying and Fault Recorder Based on Internet/Intranet. *Automation of Electric Power Systems*, **2001**, 17:39-42. [↑](#endnote-ref-29)
31. [] Jiang L, Zou Z. Information Ecology - A new paradigm of enterprise information management. Documentation, *Information & Knowledge*, **2001**, 03:2-6. [↑](#endnote-ref-30)
